# The clinical spectrum of ocular bartonellosis: a retrospective study at a tertiary centre in Malaysia

**DOI:** 10.1186/s12348-020-00224-0

**Published:** 2020-11-16

**Authors:** Michele Shi-Ying Tey, Gayathri Govindasamy, Francesca Martina Vendargon

**Affiliations:** Department of Ophthalmology, Hospital Sultanah Aminah, Johor Bahru, Johor Malaysia

**Keywords:** Ocular bartonellosis, Cat scratch disease, Bartonella hensalae, Neuroretinitis, Parinaud’s oculoglandular syndrome

## Abstract

**Background:**

Cat scratch disease (CSD) is a systemic illness caused by the gram-negative bacillus, *Bartonella henselea,* which can occasionally involve the ocular structures. The objective of this study is to evaluate the various clinical presentations of ocular bartonellosis at our institution. A retrospective review of the clinical records of 13 patients (23 eyes) with ocular manifestations of Bartonella infections over a 3-year period between January 2016 to December 2018 was undertaken at our institution.

**Results:**

The diagnosis was made based on clinical findings and in addition, with the support of the evidence of *Bartonella hensalae* IgG and/or IgM. Small retinal white lesions were the most common ocular findings in this series of patients (82.6% of eyes, 76.9% of patients). Neuroretinitis was the second most common finding (47.8% of eyes, 69.2% of patients), followed by exudative retinal detachment involving the macula (34.8% of eyes, 53.8% of patients) and Parinaud’s oculoglandular syndrome (17.4% of eyes, 23.1% of patients). Other findings like isolated optic disc oedema without macular star (8.7% of eyes, 15.4% of patients) and vitritis (4.3% of eyes, 7.7% of patients) were also observed. Ten patients (76.9%) had bilateral ocular involvement. Most of the patients were young, immunocompetent and had systemic symptoms like fever prior to their ocular symptoms. The visual acuity (VA) at initial presentation ranged from 6/6 to hand movement (mean, 6/20), and at final visit 6/6 to 6/60, (mean, 6/9). 91.7% of patients were treated with antibiotics. Only 2 patients received oral corticosteroids together with antibiotics due to very poor vision on presentation. The visual prognosis of ocular bartonellosis is generally good with 16 (88.9%) of 23 eyes having VA of 6/12 or better at final follow-up visit.

**Conclusion:**

Small foci of retinal white lesions were the most common manifestation of ocular bartonellosis in this series, followed by neuroretinitis, though an array of other ocular findings may also occur. Therefore, we should consider bartonella infection as a possible differential diagnosis in those patients.

## Background

Cat scratch disease (CSD) is a systemic illness caused by the gram-negative bacillus, *Bartonella henselea* [1]. The disease is transmitted by the bite or scratch of an infected animal, often a young cat or kitten [1]. The disease commonly affects children and young adults and is usually self-limiting in immunocompetent individuals. Typical CSD is characterized by lymphadenopathy, fever and flu-like symptoms though the pathological response to the infection can differ greatly with the status of the host immune system [2]. It may range from mild to severe, and can result in a focal suppurative response, a multifocal angioproliferative response (bacillary angiomatosis), endocarditis or meningoencephalitis, especially in the immunocompromised [2–4].

Numerous ocular manifestations of Bartonella infections can occur in those infected. The posterior segment findings reported in literature include neuroretinitis, optic neuritis, focal retinitis, choroiditis, chorioretinitis, exudative maculopathy, serous retinal detachment and vitritis [5–7]. Ocular complications like branch retinal artery occlusion, macular hole and peripapillary angiomatosis had also been described [8–10]. Conjunctival manifestations such as Parinaud’s oculoglandular syndrome and nonspecific follicular conjunctivitis were also reported [8–11].

This study aims to examine the various ocular presentation, management and visual outcome of ocular bartonellosis. In this case series, we included only patients whose clinical diagnosis of ocular bartonellosis were supported by laboratory evidence of *B. Hensalae* infection.

## Methods

We performed a retrospective review of clinical records of 13 patients (23 eyes) with ocular bartonellosis treated in Hospital Sultanah Aminah Johor Bahru, a tertiary centre in southern region of Malaysia, over a 3-year period, between January 2016 and December 2018.

The selection criteria for this study include the diagnosis of ocular bartonellosis by clinical examinations and supported by at least a single positive serology test for *B. Hensalae*. Serology tests were all performed via immunofluorescence assay (IFA) in the Bacteriology Unit, Institute for Medical Research, Ministry of Health Malaysia. The cut off value was ≥1:12 for IgM and/or ≥ 1:64 for IgG using a commercial kit to detect *Bartonella henselae* and *Bartonella quintana*.

We collected various data including demographic details, systemic comorbidities, systemic symptoms prior to ocular presentation, history of cats contact or cat scratch, *B. Hensalae* serology results, laterality, visual acuity (VA) on initial presentation and at final visit, ocular findings, spectral domain optical coherence tomography (SD-OCT) findings, follow-up duration and the treatment received by patients. The VA of patients were recorded using the standard Snellen chart at 6 m’ distance.

All patients had complete ophthalmological examination, fundus photography and SD-OCT imaging done. The clinical records and images were reviewed by trained ophthalmologists at our institution for evidence of disc oedema, macular star, retinitis or choroiditis infiltrates, intraretinal fluid (IRF), subretinal fluid (SRF) and other findings.

The study was done in accordance to the Malaysian Good Clinical Practice (MGCP) 4th edition 2018 and was registered in the National Medical Research Register (NMRR).

## Result

A total of 13 patients (23 eyes), 9 females and 4 males were diagnosed with ocular bartonellosis within the study period. The demographic data and other clinical information of our study interest are summarized in Tables 1 and 2. The mean age of presentation is 27.5 (range, 11–54 years). There were 5 (38.5%) paediatric patients (≤16 years old). Eleven patients (84.6%) had no pre-existing systemic co-morbidities. Systemic symptoms of fever, flu-like symptoms and malaise preceding the development of ocular symptoms were common, and were reported in 11 patients (84.6%). Ten patients (76.9%) were able to recall cat scratch or history of exposure to cats or kittens. In the remaining 3 patients (231%), though no definite exposure was reported, they had serological evidence of Bartonella infection. Ten patients (76.9%) presented with bilateral eye involvement, either simultaneous or sequentially, while 3 patients (23.1%) had unilateral eye involvement throughout the follow-up duration.

**Table 1:** Patients’ demographic data, systemic comorbidities, systemic symptoms, exposure to cats/kittens and serology for *B. hensalae*

Patient No., sex, age (yrs)	Systemic comorbidities	Systemic symptoms	Exposure to cats /kittens	Serology for *B. hensalae*	
1, F, 36	–	Fever × 2 wks	Yes	IgM ≥1:24 IgG ≥1:128	
2, F, 54	Diabetes, hypertension	Fever & malaise × 3 wks	Yes	IgM ≥1:24 IgG ≥1:128	
3, M, 28	–	-	Yes	IgM ≥1:24 IgG ≥1:128	
4, F, 12	–	Fever × 2 wks	Yes	IgM ≥1:24 IgG ≥1:128	
5, F, 50	–	Fever × 1 wk	Unsure	IgM ≥1:24 IgG ≥1:128	
6, M, 11	–	Fever & flu-like symptoms × 1 wk	Yes	IgM ≥1:24 IgG ≥1:128	
7, F, 27	–	Fever & flu-like symptoms × 1 wk	Yes	IgM ≥1:24 IgG ≥1:128	
8, F, 12	–	Fever × 5 days, left submandibular & pre-auricular lymphadenopathy	Yes	IgM ≥1:24 IgG ≥1:128	
9, F, 38	Renal failure	-	Unsure	IgM ≥1:12 IgG ≥1:64	Repeated: IgM ≥1:12 IgG ≥1:256
10, F, 11	–	Fever × 1 wk., left pre-auricular & cervical lymphadenopathy	Yes	IgM < 1:12 (negative) IgG ≥1:128	
11, M, 15	–	Fever × 5 days, right submandibular lymphadenopathy	Yes	IgM ≥ 1:24 IgG < 1:64 (negative)	
12, F, 34	–	Fever × 1 wk	Unsure	IgM < 1:12 (negative) IgG ≥1:64	Repeated: IgM < 1:12 IgG ≥1:256
13, M, 30	–	Fever × 1 wk	Yes	IgG ≥ 1:24 IgM ≥ 1:128	

*F* Female, *M* Male

**Table 2:** Laterality, initial and final VA, ocular manifestations, OCT findings, follow-up duration and treatment

Patient No.	Lat	Eye	Initial VA	Final VA	Ocular manifestations	OCT findings	Follow up	Treatment
1	BE	OS OD	6/7.5 6/20	6/6 6/6	LE neuroretinits & flame haemorrhage (at optic disc margin), BE small retinal white lesions	LE SRF under macula & hyperreflective foci within OPL (HE)	23 wks	Doxycycline × 6wks
2	BE	OS OD	6/60 6/6	6/60 6/7.5	RE neuroretinitis, LE flame haemorrhage (at optic disc margin), BE small retinal white lesions	RE SRF under macula & hyperreflective foci within OPL (HE)	16 wks	Ciprofloxacin × 5 wks Oral Prednisolone × 1mo
3	BE	OS OD	6/15 6/6	–	RE neuroretinitis & flame haemorrhage (at optic disc margin), LE one small flame haemorrhage at supratemporal arcade, BE small retina white lesions	RE hyperreflective foci within OPL (HE), IRF (CMO) & SRF under macula	–	Doxycycline × 6 wks
4	BE	OS OD	CF 6/9	6/9 6/6	RE neuroretinitis, small flame haemorrhage (at optic disc margin) with marked retinal vessels tortuosity & dilatation, BE small retinal white lesions	RE SRF under macula & hyperreflective foci within OPL (HE)	9 wks	Doxycycline × 9 days Azithromycin ×4 wks Oral Prednisolone × 1 mo
5	BE	OS OD	6/6 6/12	6/6 6/6	LE neuroretinitis, BE small retinal white lesions	RE hyperreflective RNFL foci (retinitis), hyperreflective foci within OPL (HE) & IRF	7 wks	Ciprofloxacin ×3 days Augmentin × 2wks
6	BE	OS OD	6/30 HM	- -	BE neuroretinitis and flame haemorrhages (at BE optic disc margin), BE small retina white lesions with marked retinal vessels tortuosity & dilatation (L > R)	BE SRF under macula (L > R) & hyperreflective foci within OPL (HE)	–	Doxycycline × 2 wks
7	BE	OS OD	1/60 6/9	6/18 6/9	RE neuroretinitis, flame haemorrhage (at optic disc margin) with marked retinal vessels tortuosity & dilatation, BE small retinal white lesions	RE IRF (CMO), SRF under macula & hyperreflective foci within OPL (HE)	6 wks	Doxycycline × 6 wks
8	BE	OS OD	6/9 6/9	6/9 6/9	BE Large granulomatous lesions at palpebral conjunctivae	–	16 wks	Erythromycin × 2 wks
9	BE	OS OD	6/9 6/9	6/9 6/9	BE neuroretinitis (partial macular star) & BE small retinal white lesions	BE hyperreflective foci within OPL (HE)	36 wks	–
10	LE	OS OD	6/9 6/9	–	LE granulomatous follicular conjunctivitis	–	–	Doxycycline × 1 wk
11	RE	OS OD	6/6 6/6	6/6 6/6	RE granulomatous follicular conjunctivitis	–	2 wks	Doxycycline × 1 wk
12	LE	OS OD	6/6 6/9	6/6 6/9	LE optic disc swelling with vitritis & LE small retinal white lesions	LE PVD with hyperreflective spots in vitreous (vitritis) & swollen LE ONH	5 wks	Ciprofloxacin × 1 mo
13	BE	OS OD	6/6 6/38	6/6 6/6	LE neuroretinitis, BE small retinal white lesions	LE SRF under macula & BE hyperreflective foci within OPL (HE)	8 wks	Doxycycline × 6 wks

*Lat* Laterality, *BE* Both eyes, *RE* Right eye, *LE* Left eye, *OS* Right eye, *OD* Left eye, *CF* Counting finger, *HM* Hand movement, *OCT* Optical coherence tomography, *SRF* Subretinal fluid, *OPL* Outer plexiform layer, *HE* Hard exudate, *CMO* Cystoid macular oedema, *IRF* Intraretinal fluid, *PVD* {posterior vitreous detachment, *ONH* Optic nerve head

The mean follow-up time was 12.8 weeks (range, 2–36 weeks). Three patients were lost to follow-up and excluded from final VA analysis. The initial presenting VA ranged from 6/6 to hand movement (mean 6/20) and at final follow-up visit 6/6 to 6/60 (mean 6/9), as shown in Table 2. Twelve of the 13 patients (92.3%) were treated with systemic antibiotics. Eight patients (61.5%) were treated with doxycycline, three patients (23.1%) with ciprofloxacin (23.1%), one patient (7.7%) with azithromycin and one patient (7.7%) with erythromycin. Two patients had switched antibiotics during the course of the treatment due to suspected adverse reactions to the initial antibiotics. The average duration of systemic antibiotics were 4–6 weeks for posterior segment involvement and 1–2 weeks for Parinaud’s oculoglandular syndrome. In addition, two patients (15.4%) were treated with oral corticosteroids due to very poor presenting vision. The visual prognosis of ocular bartonellosis is generally good with 16 (88.9%) of 23 eyes having VA of 6/12 or better at final follow-up visit (excluding those who were lost to follow-up).

**Fig. 1:**
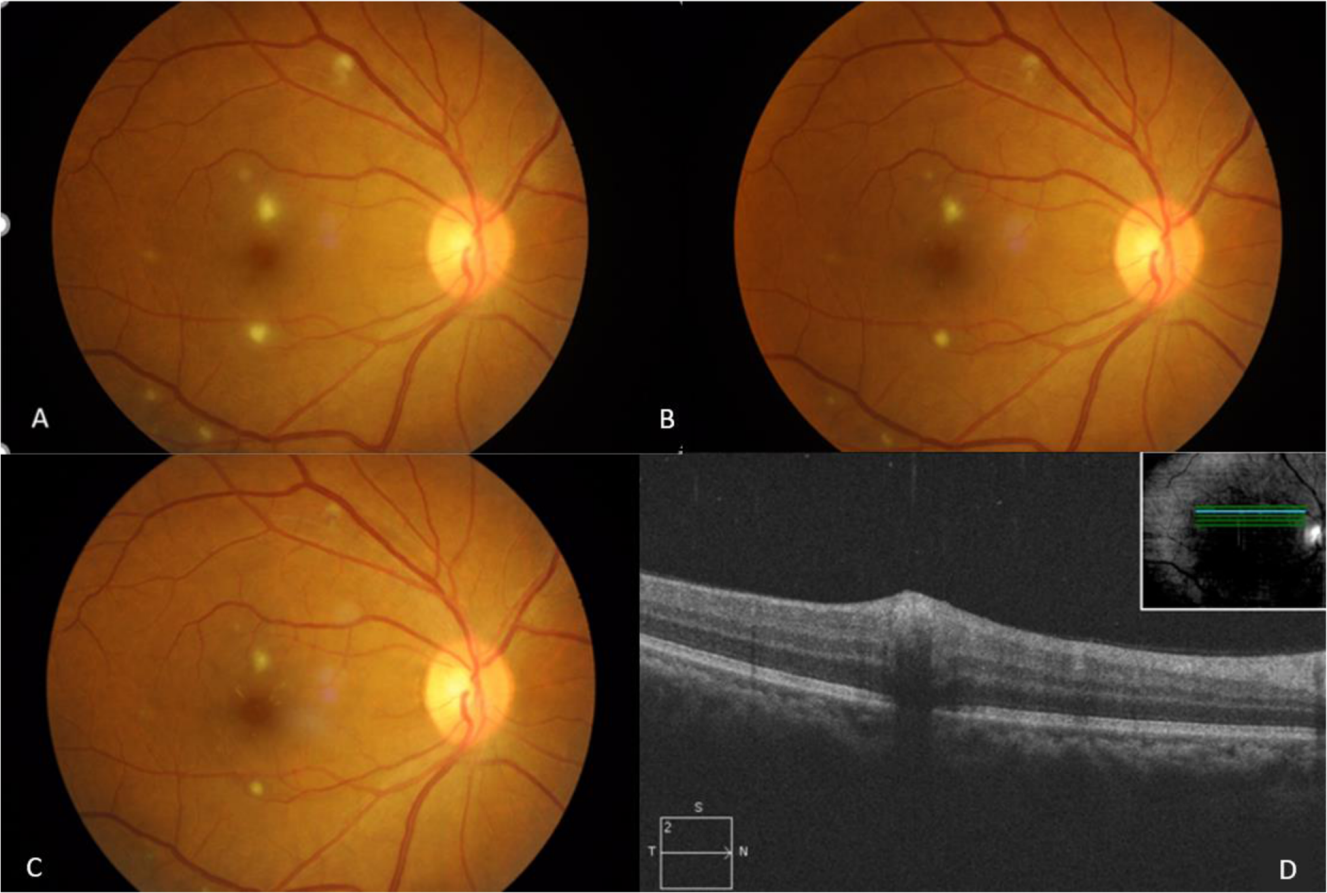
Patient no. 5. **a**, **b** and **c** Colour fundus photograph of the right eye: multiple small foci of yellow-white retinitis lesions. The lesions’ borders become more well-defined and the sizes reduce over time. **d** SD-OCT of the same eye: cross section superior to the fovea across the foci of retinitis appear as hyperreflectivity in the inner retinal layers while casting a shadow below it

**Fig. 2:**
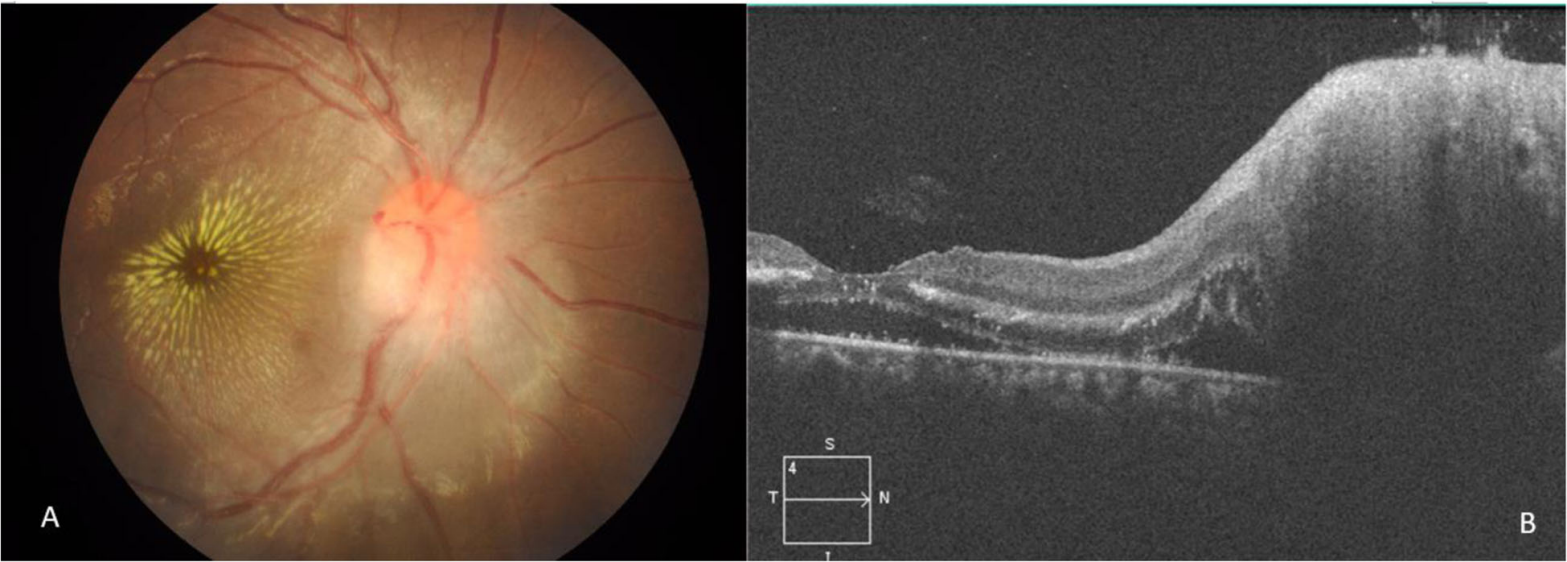
Patient no. 4. **a** Colour fundus photograph of the right eye: peripapillary retinal oedema with prominent macular star (typical neuroretinitis), splinter haemorrhage near the margin of the disc with tortuous and markedly dilated retinal vessels. **b** SD-OCT of the same eye: showing swollen ONH with SRF causing exudative retinal detachment involving the fovea. Hyperreflective spots within the OPL correspond to the macular star clinically

**Fig. 3:**
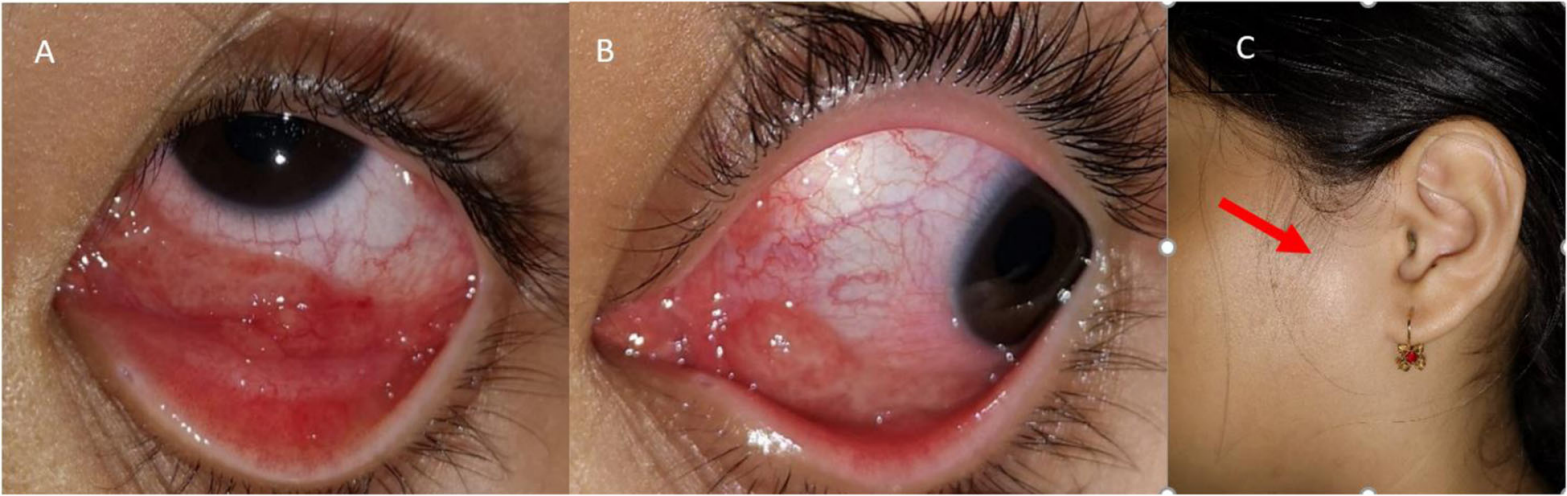
Patient no. 10. A 11-year old girl with history of cat scratch presented with left eye redness, fever, cervical and pre-auricular lymph nodes swelling. **a** and **b** Granulomatous nodules on left lower tarsal and bulbar conjunctivae. **c** Red arrow showing pre-auricular lymph node enlargement

Ocular findings of our patients are listed in Table 2. Ten out of the 13 patients (76.9%) presented with posterior segment manifestations, while 3 out of the 13 patients (23.1%) had Parinaud’s oculoglandular syndrome without posterior segment involvement. Small foci of retinal white lesions were the most common manifestations, occurring in 19 eyes (82.6%) (Fig. 1a-d). Neuroretinitis was the second most common finding, seen in 11 eyes (47.8%) (Fig. 2a-b). This is followed by exudative retinal detachment (SRF under fovea evident on SD-OCT) in 8 eyes (34.8%) and Parinaud’s oculoglandular syndrome in 4 eyes (17.4%) (Fig. 3a-c). Other less common findings were isolated optic disc oedema without macular star, occurring in 2 eyes (8.7%) and vitritis which occurred in 1 eye (4.3%). SD-OCT imaging was done for all the eyes with posterior segment involvement, and it was found that among them, 13 eyes (68.4%) have hyperreflective spots within the outer plexiform layer (OPL), corresponding to the stellate macular hard exudates seen clinically and 8 eyes (42.1%) have SRF under fovea causing serous elevation of the neurosensory retina i.e. exudative retinal detachment.

## Discussion

Similar to earlier retrospective studies, the patients in our series were young (mean, 27.5 years) [9, 12, 13]. Simultaneous or sequential bilateral eye involvement were more common in our series (76.9% of patients). This is in contrast to prior reports, wherein unilateral eye involvement was reported to be more common [1, 5]. Though there were 3 patients who were unsure if they had contact with cats or kittens prior to the ocular presentation, they had clinical and serological evidence of bartonella infection. Some reports have shown that apart from cats, animals like dogs, monkeys and porcupines may harbour the agent [14]. Cat fleas *(Ctenocephalides felis)* may also play a role as an arthropod vector [14].

**Fig. 4:**
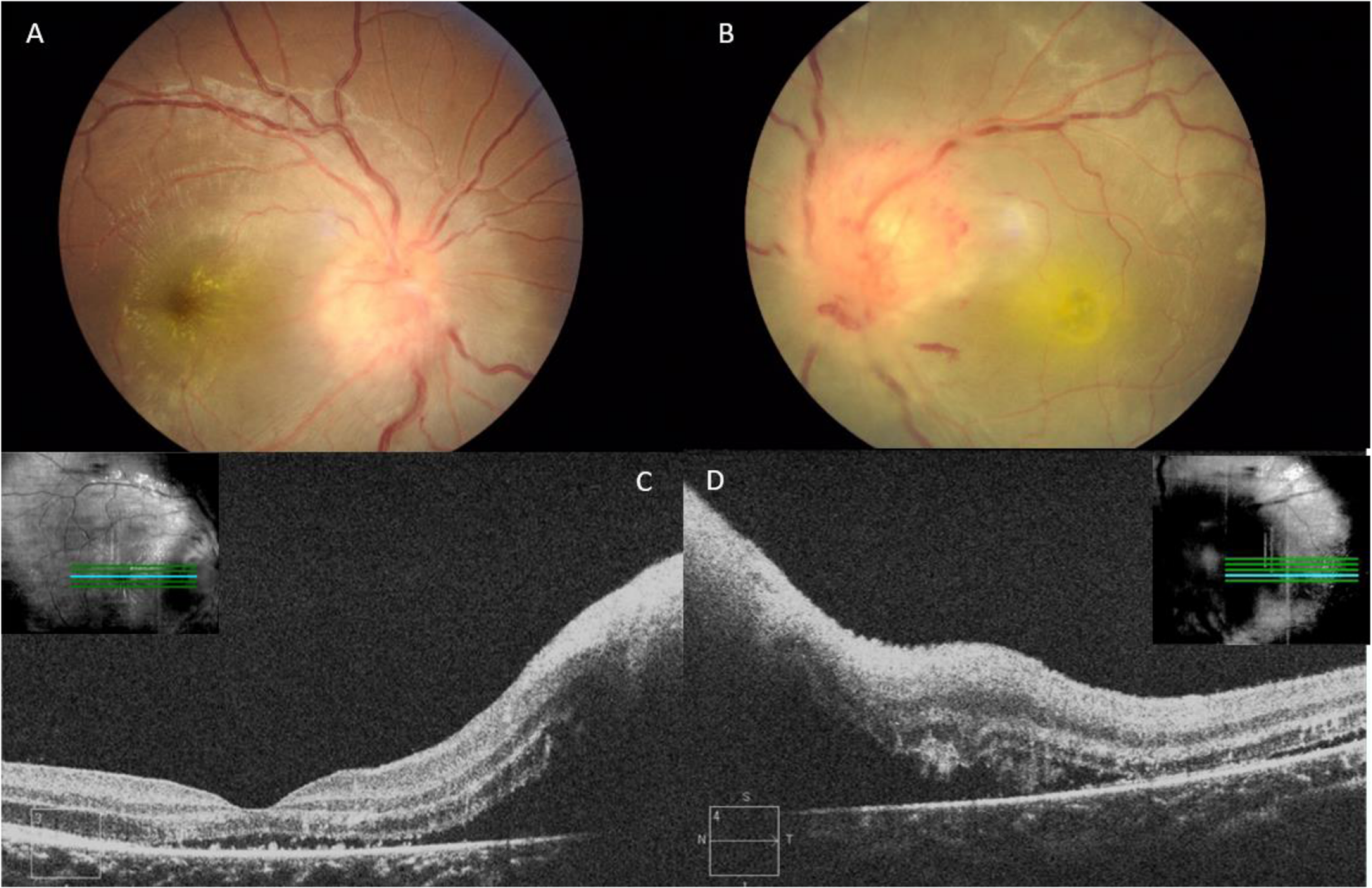
Patient no 6. **a** and **b** Colour fundus photograph of the right and left eye respectively: both eyes have optic disc oedema with yellowish macular exudation (forming bilateral neuroretinitis) and also marked venous engorgement and tortuosity. **c** and **d** Corresponding SD-OCT macula of the right eye and left eye: fluid tracking from the oedematous optic disc to the subretinal space. Left eye especially, exhibits a loss of the normal foveal contour and marked serous macular detachment

The ocular findings in our series of patients are similar to the one reported by Solley et al. (1999) which described retinal or choroidal white lesions to be the primary ocular manifestations of CSD and neuroretinitis as the second most common ocular presentation [1]. However, there are studies that reported neuroretinitis as the commonest ocular manifestation, such as the one by Ormerod et al. (1999) [8]. Neuroretinitis is a vasculitic process within the optic nerve head coupled with secondary stellate macular exudates [15]. The optic disc swelling is known to precede stellate exudation by 2–4 weeks [9]. There have been few reported cases of bilateral neuroretinitis [15, 16]. In our series, only 2 patients had the rare presentation of bilateral neuroretinitis (Fig. 4a-d), similar to several other studies that had reported neuroretinitis to be commonly unilateral [5, 8]. We observed in our series that 7 of 10 patients (70%) with posterior segment manifestation of ocular bartonellosis presents with neuroretinitis in one eye, coupled with small foci of white retinal or choroidal lesions in both of the eyes. In addition, eight eyes (34.8%) was found to have exudative retinal detachment, evident by presence of SRF under fovea on SD-OCT (Figs. 2b, 4c-d and 6b, d). Peripapillary serous retinal detachment as a result of optic disc oedema in Bartonella patients has been reported to be common yet underrecognized [17]. Furthermore, six patients had flame-shaped haemorrhages at the margin of the oedematous optic disc. Habot-Wilner Z et al. (2011) had reported clusters of telangiectatic vessels on the optic nerve edge with hyper-fluorescence and leakage when fluorescein angiogram was performed [18]. These findings may be explained by the predilection of *B. henselae* towards the vascular endothelium [19]. Likewise, patients with markedly swollen optic disc tend to have more tortuous and dilated retinal vessels and correspondingly more extensive macular exudates, SRF and poorer VA (Fig. 4a-b). Collectively, these findings provide clues to help the clinician in diagnosing an ocular bartonella infection.

**Fig. 5:**
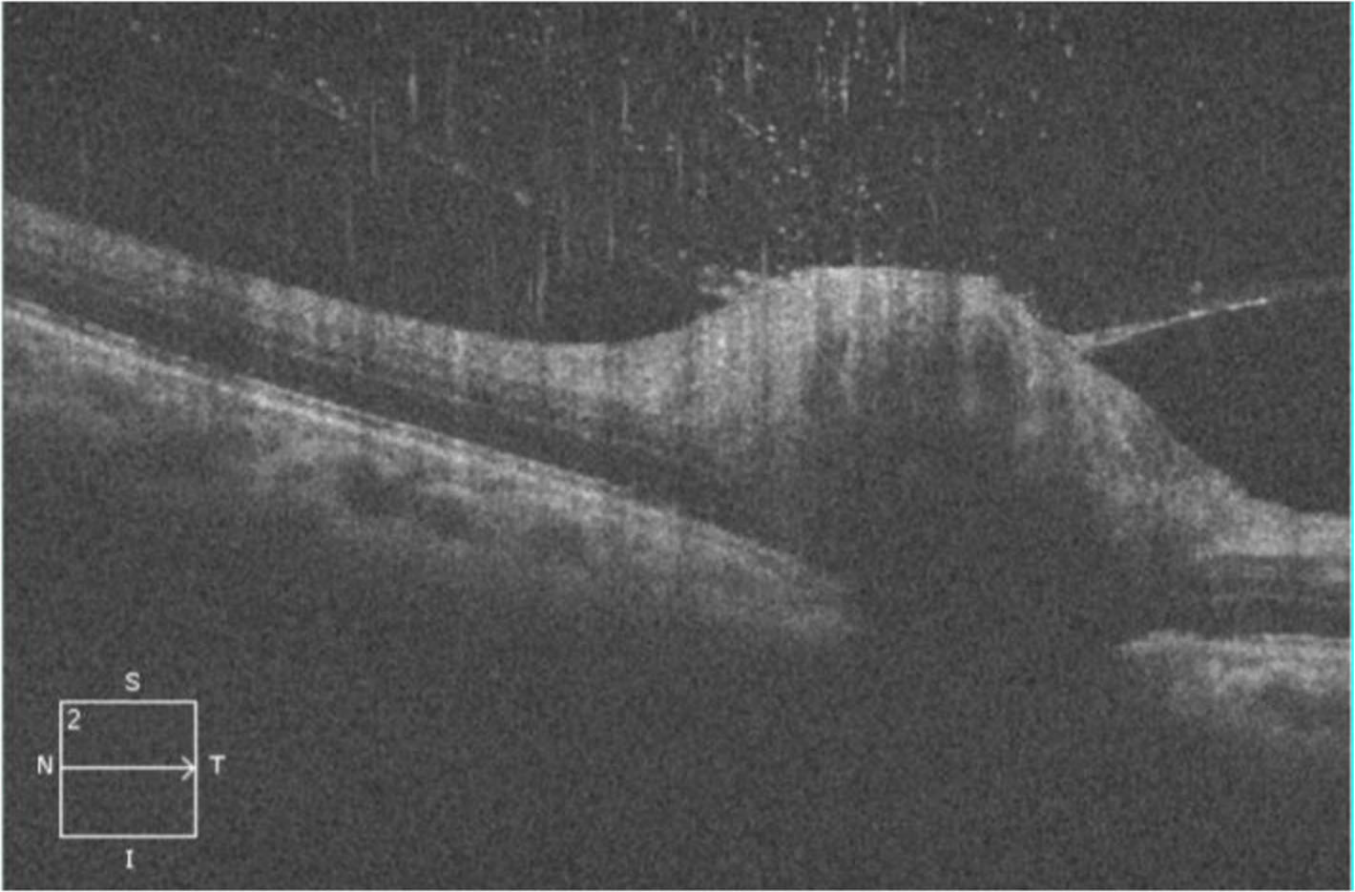
Patient no. 12. SD-OCT of the left eye: swollen ONH with overlying vitritis and incomplete posterior vitreous detachment (PVD). No SRF or macula star developed in this patient. The ONH oedema and vitritis resolve following treatment

**Fig. 6:**
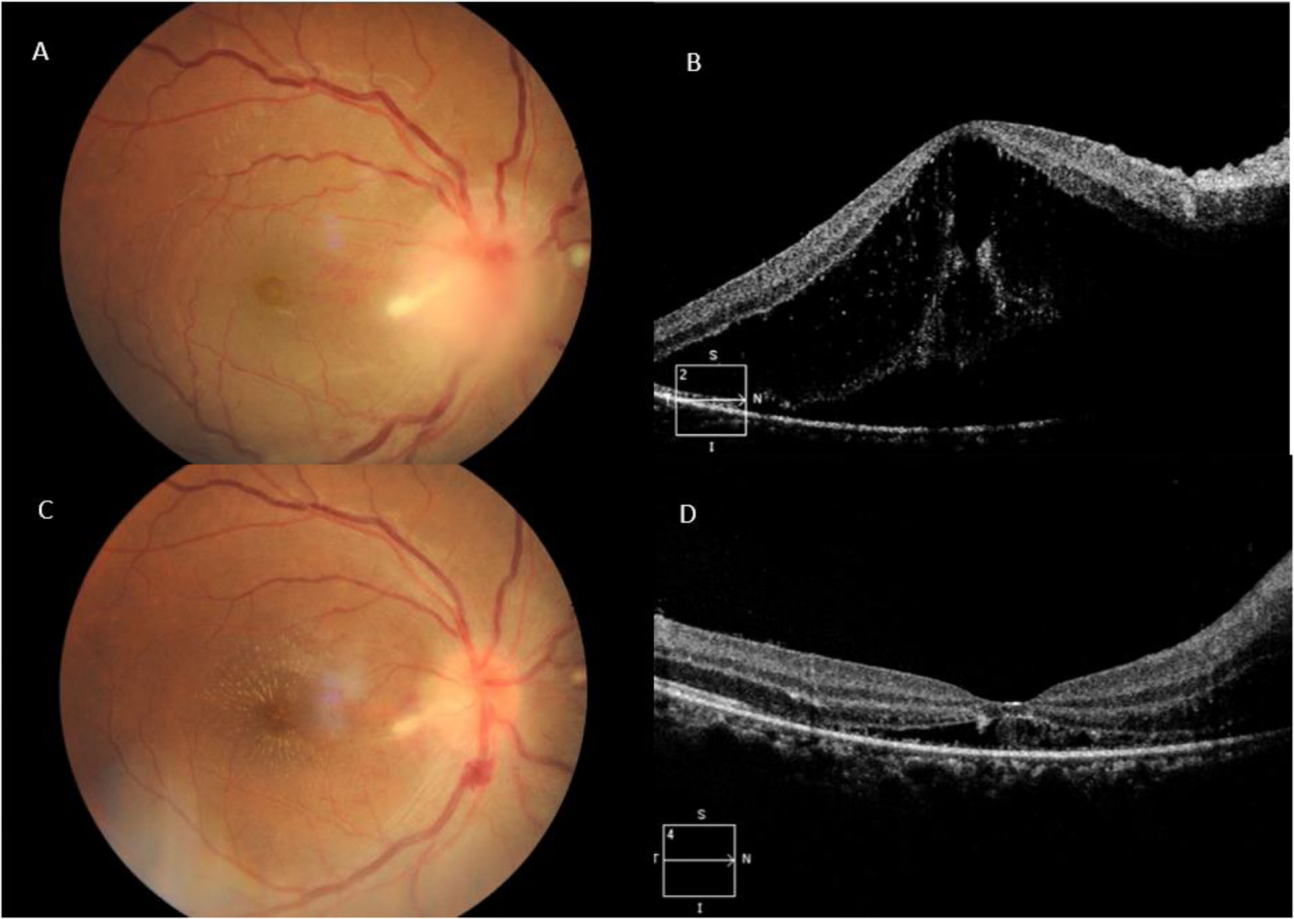
Patient no. 7. **a** Colour fundus photograph of the right eye on initial presentation: swollen optic disc, macula oedema, peripapillary RNFL haemorrhages, yellow-white deep retinal lesions near the disc and venous engorgement. VA was 1/60. **b** Corresponding SD-OCT of the right eye: prominent IRF and SRF with loss of foveal contour. c Colour fundus photograph of the same eye after 2 weeks: resolving optic disc oedema and decrease in venous engorgement and tortuosity. **d** Corresponding SD-OCT of the same eye 2 weeks later: restoration of the foveal contour, IRF has resorbed with some residual SRF. VA improved to 6/18

Of note, several cases showed interesting findings. Patient no. 6 had massive optic nerve head swelling with substantial SRF that caused marked serous detachment of the macular (Fig. 4a-d). The fundamental pathophysiology of this occurrence has been described as transudation from the inflamed optic nerve head (ONH) into the subretinal space of an apparently normal macula [19]. On the other hand, patient no. 12 had optic disc oedema without macular star throughout the follow-up period. This particular patient was also the only one who manifested subclinical vitritis as evident on the SD-OCT image (Fig. 5), besides having the typical small foci of white retinal infiltrates in that same eye. Other possible infections like toxoplasmosis, syphilis, tuberculosis and endogenous endophthalmitis were ruled out in this particular patient. Thus, the absence of a neuroretinitis (or in this case, an optic disc swelling without macular involvement) does not safely exclude the possibility of ocular bartonellosis. Likewise, when a patient presents with injected conjunctivae and granulomatous follicular conjunctivitis, one should consider the possible diagnosis of Parinaud’s oculoglandular syndrome, particularly if the patients had reported contact with cats or kittens. In our series, we report 3 patients who presented with Parinaud’s oculoglandular syndrome (Fig. 3a-c). All of them had fever prior to their ocular symptoms and all had palpable enlargement of the ipsilateral cervical lymph nodes. Hence, examining patients’ cervical lymph nodes for lymphadenopathy and exploring symptoms of preceding fever and systemic symptoms may aid in diagnosing Parinaud’s oculoglandular syndrome. It is also worth noting that patients with Parinaud’s oculoglandular syndrome were of the younger age group in our series, between 11 and 15 years old. Parinaud’s oculoglandular syndrome is a frequently-missed diagnosis and likely remains significantly under-reported [20]. Patients may not seek treatment due to the self-limiting nature of the disease, good response to empirical antibiotic treatment and little effect on vision as compared to the posterior segment presentations. In our series, all 3 patients with Parinaud’s oculoglandular syndrome had complete resolution of symptoms by the end of their follow-up visit. Ocular manifestation of vaso-occlusive events like retinal artery or retinal vein occlusions were reported in some studies, but none of our patients had such presentation [1, 5, 18]. Anterior segment inflammation has also been described [13, 21]. Nevertheless, it did not occur in any of our patients. Patients who had posterior segment manifestations in our series showed typical macular changes on SD-OCT which includes hyperreflective foci within the retinal nerve fibre layer (RNFL) (retinitis spots clinically), hyperreflective foci of retinal exudates within the OPL (hard exudates clinically), presence of SRF tracking from the swollen ONH to the sub-foveal space (exudative retinal detachment at the sub-foveal/macular region) and IRF causing retinal thickening and loss of foveal contour (Figs. 1d, 2b, 4c-d and 6b, d).

Antimicrobial treatment for *B. Hensalae* infection in the immunocompetent individuals have been controversial and no clear definitive treatment recommendations exist [8, 9]. The difficulty to establish a treatment recommendation is partly due to the Bartonella infection being generally self-limiting [13, 21]. Prior reports have suggested that early antimicrobial treatment may speed recovery and improve the final visual outcome [21, 22]. In our study, most patients (92.3%) were treated with systemic antimicrobial, except for patient no. 9 as she was initially treated as ocular tuberculosis as her Mantoux test was borderline. She developed stellate macula exudates later on in association with optic disc oedema, which raised the suspicion of Bartonella infection and this was confirm by a 4-fold increase in *B. Hensalae* serology titres on repeated testing. However, by the time the second serology result was available, her vision has already improved to 6/9, hence antibiotic treatment was not commenced. Rifampicin, gentamicin, cotrimoxazole, ciprofloxacin, and doxycycline have shown efficacy in the treatment of bartonella infection [21]. Some have reported azithromycin as the preferred antimicrobial of choice in treating ocular bartonellosis as it has better compliance, easier once daily dosing and better safety profile compared to doxycycline which is given twice a day and tends to cause gastrointestinal upset [22]. Also, azithromycin is also the only agent studied in a randomized controlled study for CSD [22]. Nevertheless, doxycycline continued to be used widely for the treatment of ocular bartonellosis in many centres and in our series, doxycycline is used most frequently, in 61.5% of patients. This is in part due to the ability of doxycycline to adequately penetrate the tissues of the eye and central nervous system and also since it is more widely available in our hospital setting [9]. The role of systemic corticosteroids is also debatable and not clearly established, often used in those with very poor vision on presentation. The association between visual outcome and systemic corticosteroids use was reported in a few studies. Chi et al. (2016) reported no association between systemic corticosteroids use and visual outcome [5] . Meanwhile, a multicentre retrospective cohort study by Habot-Wilner Z et al. (2011) reported that combined antimicrobial and systemic corticosteroids treatment was associated with a better visual outcome than regimen of antibiotics alone in patients with VA worse than 6/9 at presentation [18]. In a case series of 14 patients in Japan reported by Kodoma et al, 13 of them received systemic steroids and 2 of them were treated with 1000mg of methylprednisolone pulse therapy [23]. In our series, systemic corticosteroids were commenced in patients who had significantly poor vision on presentation, i.e. VA of 6/60 or less. Overall, 16 eyes (88.9%) achieved a VA of 6/12 or better at final follow-up visit. One patient had a final VA of 6/60 due to an atrophic macula following resolution of SRF in the same area and another one had a final VA of 6/18 due to residual macula star (the patient did not turn up for her subsequent follow-up visit after the 6th week and we were unable to ascertain whether the VA continue to improved later on).

There are few limitations to our study. Firstly, a second sample of serology should ideally be sent 2 weeks after the initial sample, as a 4-fold increase in titer is a definite diagnostic of *B. Hensale* infection. In our series, only two patients (patient no. 9 and patient no. 12) had a second Bartonella serology sample taken. In our clinical setting, a second sample is not usually taken as the results generally take up to 6 weeks. Most patients would have already responded to the treatment given or their symptoms may have resolved spontaneously by then. Therefore, further testing neither have aided in diagnosis nor altered clinical decision making. Also, some of the patients presented late to the clinic, and as such had missed the optimum window period for testing that would yield peak blood levels of IgM and IgG. In IFA, IgM positivity has been reported to decrease after 8 weeks while IgG positivity peaked after 6–8 weeks. In addition, the IFA used in our bacteriology unit measures antibodies to both *Bartonella henselae* and *Bartonella quintana*; therefore, we were unable to differentiate between these *Bartonella* spp. infections. The polymerase chain reaction test is an alternative method to test for the *Bartonella* spp. and is highly sensitive but is not routinely done in our hospital setting due to the cost involved [24]. Secondly, it must be remembered that the true frequency of the ocular findings in ocular bartonellosis might differ from our results due to the small sample size of our study. Nevertheless, the main purpose of this study is not to precisely quantify the true incidence of each finding but rather to highlight some of the less, well appreciated clinical findings of ocular bartonellosis. Thirdly, this study was not able to establish association between antibiotics and corticosteroids use and visual outcome due to the retrospective design and lack of control group arm. In order to determine whether antibiotics and corticosteroids treatment is helpful in improving VA in patients who present with ocular manifestations of Bartonella infection, a randomized clinical trial comparing a standardized treatment regimen to placebo will be necessary.

## Conclusion

Small foci of retinal white lesions were the most common ocular manifestation of ocular bartonellosis in this study, followed by neuroretinitis, though patients can also present with other ocular signs as described above. Most of the patients who were affected were immunocompetent, had bilateral eye involvement and had fever prior to their ocular presentations. The visual prognosis of ocular bartonellosis is generally good.

## Data Availability

Please contact authors for data requests.

## Abbreviations

CSD: Cat scratch disease
VA: Visual acuity
IFA: Immunofluorescence assay
SD-OCT: Spectral domain optical coherence tomography
CMO: Cystoid macular oedema
RAPD: Relative afferent pupillary defect
IRF: Intraretinal fluid
SRF: Subretinal fluid
MGCP: Malaysian Good Clinical Practice
NMRR: National Medical Research Register
OPL: Outer plexiform layer
ONH: Optic nerve head
RNFL: Retinal nerve fiber layer
